# Benefit of the N-of-1 Approach Versus Aggregate Analysis in Tracking Individual Trajectories During Pregnancy: Comparison of Longitudinal Wearable Observational Studies

**DOI:** 10.2196/86203

**Published:** 2026-04-28

**Authors:** Tina Behrouzi, Jennifer Yu, Robin Yang, Adrien Boch, Anna Goldenberg, Sarah M Goodday, Stephen H Friend

**Affiliations:** 1 Department of Computer Sciences, University of Toronto, Toronto, ON, Canada; 2 Vector Institute, Toronto, ON, Canada; 3 Genetics and Genome Biology Program, Hospital for Sick Children, Toronto, ON, Canada; 4 Tri-institutional Computational Biology & Medicine, Weill Cornell Medicine, New York, NY, United States; 5 4YouandMe, 185 Great Neck Road, Great Neck, NY, 11021-3326, United States, 1 215-421-3073; 6 Meta (United States), San Francisco, CA, United States; 7 Evidation Health (United States), San Mateo, CA, United States; 8 Department of Laboratory Pathology and Medicine, University of Toronto, Toronto, ON, Canada; 9 Canadian Institute for Advanced Research, Toronto, ON, Canada; 10 Department of Psychiatry, University of Oxford, Oxford, United Kingdom

**Keywords:** digital health technologies and wearables, N-of-1 analysis and personalized insights, pregnancy, maternal health, advanced statistical modeling in health care

## Abstract

**Background:**

Personal digital health technologies (DHTs) enable real-time monitoring of physiological metrics and behavioral data, including heart rate variability (HRV), supporting analysis of pregnancy-related conditions and personalized care throughout the perinatal period. While recent studies demonstrate the utility of personal DHTs in tracking pregnancy-related symptoms, they often rely on aggregate statistical methods that overlook individual variability.

**Objective:**

This study aims to compare aggregate and individual-level analyses of DHT data for pregnancy-related conditions, using the comprehensive BUMP (Better Understanding the Metamorphosis of Pregnancy) dataset to highlight the importance of individual variability and data heterogeneity.

**Methods:**

We analyzed wearable and self-reported data from 256 participants enrolled in the BUMP study (January 2021 to May 2022), including HRV, sleep, and fatigue measured via Oura Rings and smartphone surveys. Individual-level (N-of-1) trajectories were evaluated and compared with aggregate results to uncover personal and collective trends. A statistical method was developed to assess the influence of adverse events and severe symptoms, while case studies explored confounding and modifying factors underlying heterogeneity. Comprehensive statistical analysis included the coefficient of determination, Kolmogorov-Smirnov tests, likelihood ratio tests, and Welch *t* tests, with interindividual variability flagged based on high-variability thresholds.

**Results:**

Substantial interindividual variability was observed across all features. Only 4.76% (12/256) of participants exhibited an HRV inflection at the aggregate week-33 inflection point, with a coefficient of variation of 14.24%. The median value of the gestational week in individual fatigue troughs was 23 (IQR 8; range 8-38) weeks, differing from aggregate estimates. Distributional comparisons showed no statistically significant differences in individual-level model fit (*R*²) by pregnancy complications or age (*P* values ranging from .06 to .99 across all model fit comparisons). Case studies further highlighted both intraindividual and interindividual differences, emphasizing the importance of considering external factors, such as adverse events and severe symptoms.

**Conclusions:**

Our findings show that aggregate wearable data often fail to generalize across populations, oversimplifying pregnancy-related physiological and subjective changes. This simplification can obscure individual trajectories, leading to generalized insights that may not reflect many pregnant women’s experiences. Our results highlight the impact of heterogeneity on pregnancy outcomes, emphasizing the need to move beyond one-size-fits-all models and leverage DHT for personalized care.

## Introduction

Pregnancy is a critical period marked by significant physiological and psychological changes, influencing maternal and fetal health [1]. Conditions such as gestational hypertension, preeclampsia, preterm birth, and postpartum depression impose significant global health burdens [2]. Despite their prevalence, there is still a limited understanding of the underlying factors driving pregnancy outcomes and an essential need for improved support systems to address women’s health during this critical transition [3].

Wearable devices, digital health apps, and other remote smart devices (personal digital health technologies [DHTs] [4]) are increasingly recognized for their potential to transform health care [5,6]. DHTs enable the semicontinuous, real-time monitoring of physiological measures and behavior information, including cardiovascular metrics, activity levels, sleep patterns, and high-frequency subjective symptoms [7-9], particularly relevant to pregnancy. These approaches offer an advantage over traditional aperiodic, symptom-monitoring methods in capturing subtle individual differences in a remote setting, filling in the gaps between clinic visits, and providing tools that could track key symptoms in populations who lack access to prenatal care [10,11]. Harnessing data from diverse DHTs could enhance early detection of pregnancy-related conditions [4,12,13] and enable personalized care throughout the perinatal period [14].

While recent studies have demonstrated the value of personal DHTs in tracking pregnancy-related symptoms and outcomes [15], they often rely on aggregate statistical methods [14,16-18] that can overlook individual variability, as a one-size-fits-all approach may not apply effectively to every individual [19]. A recent large-scale longitudinal study [20] demonstrates systematic deviations of sleep metrics from individual prepregnancy baselines across pregnancy and postpartum, providing population-level context in the field. However, variability in individual behaviors and physiological responses, along with limitations such as small sample sizes in some studies, continues to challenge the generalizability of population-based models. Personal DHTs in pregnancy offer individualized risk monitoring, but recent studies rely on aggregated data instead of N-of-1 analyses. Closing this gap is key to making N-of-1 a standard and maximizing wearable data in future research.

This paper aims to compare the generalizability of personal DHT study results derived from aggregate data with those based on N-of-1 analyses in pregnant individuals using data from a US-based digital health pregnancy study (the BUMP [Better Understanding the Metamorphosis of Pregnancy] study [21]). Three recently published pregnancy digital health studies [16-18] were selected that focused on 3 key measures: heart rate variability (HRV) [16], sleep [18], and fatigue [17]. Aggregated results from these studies were replicated using the BUMP study dataset and were extended by exploring N-of-1 analyses of these key measures. By applying N-of-1 methods, including spline fitting, pregnancy condition analysis, and analyzing certain women in case studies, we aimed to dissect individual differences often overlooked in aggregated approaches.

## Methods

### Study Design

The BUMP study was a participant-centric digital health study that tracked maternal symptoms using wearable devices and smartphone apps from preconception, through pregnancy, and up to 3 months postpartum [21]. The study was conducted fully remotely and designed to capture high-frequency physiological and self-reported data across the perinatal period. The present analysis focused on 3 key features, including HRV, sleep (deep sleep and awake time), and fatigue, drawn from multimodal data collected through devices like the Oura Ring (Oura Health Inc) [22]. These features were chosen to capture both objective and subjective measures of health and align with recently published maternal health wearable studies. Nighttime HRV was calculated using the root mean square of successive differences between normal heartbeats method from 5-minute interval data collected by the Oura Ring. Awake time and deep sleep were derived from the Oura Ring’s 3D accelerometer measurements and 5-minute sleep rankings, respectively. Fatigue was a self-reported feature tracked daily via the BUMP study smartphone app.

### Recruitment

Participants were recruited through systematic, multichannel digital outreach strategies as described in the published BUMP study protocol [21]. Recruitment occurred primarily via a secure patient-provider platform operated by Sema4, through which potentially eligible individuals received study invitations. Additional recruitment efforts included targeted outreach through digital advertisements and social media platforms to broaden reach and ensure diverse participation.

### Ethical Considerations

The BUMP study was approved by the Advarra Institutional Review Board (Pro00047893). Electronic informed consent was obtained from all participants through the study application prior to any data collection. Participant privacy and confidentiality were protected through data deidentification and secure data storage practices. Access to coded study data is restricted and governed through controlled-access platforms in accordance with institutional and regulatory requirements. Participant compensation, where applicable, was provided as outlined in the approved study protocol.

### Participants

Out of 431 participants, 275 were included after excluding those with more than 60% missing data, missing delivery information, or fewer than 30 data points. The number of participants in each complication group was as follows: 37 with preterm birth, 42 with gestational diabetes mellitus, 38 with preeclampsia, 66 with gestational hypertension, 39 with postpartum hemorrhage, 189 with major depression, and 17 were categorized as healthy individuals (as defined as normal weight [BMI<25], age<35 years, and no complications during pregnancy and postpartum). Further demographic information is provided in Table S4 in Multimedia Appendix 1.

### Data Preprocessing

Details on data collection and study measures are in Multimedia Appendix 1. We used aggregated daily summaries from 5-minute interval sleep data, calculating total duration for each sleep stage (deep sleep, awake, and rapid eye movement) across the sleep period. Moreover, HRV was averaged over the entire sleep period. Duplicated time points for objective features were removed. Outliers were identified using the 0.05 and 0.95 quantiles estimated from a randomly selected subset of 100 participants from the study population; these quantile thresholds were then applied uniformly across all participants and time points, with values outside the range set to not a number. For quadratic analysis, data were smoothed by gestational week, and averages were computed. Data preprocessing involved calculating the number of days relative to each participant’s delivery date (days since delivery) at every sample point and determining the corresponding gestational age. Gestational age was calculated based on the estimated due date provided during the initial enrollment and adjusted according to the actual birth date when necessary. This information was crucial in aligning wearable device data and survey responses with the appropriate stage of pregnancy for accurate longitudinal analysis. Fatigue was kept in its continuous form between 1 and 7 values where 1 is “none” and 7 is “severe.”

A case study analysis examined factors contributing to heterogeneity and varying signal behavior among participants, including self-reported severe daily events, severe symptom events, and adverse event (AE) reports. Severe symptoms, outlined in Table S2 in Multimedia Appendix 1, were reported biweekly. To enhance the evaluation of their impact on distribution change analysis, we applied the SentenceTransformer [23] model to generate embeddings for daily and biweekly features, enabling a more precise estimation of severe symptom timestamps.

Expanding this analysis, we examined the impact of AEs on feature trajectories using a dataset subset with free-text descriptions recorded by engagement experts after biweekly check-ins. To ensure consistency and extract insights, we used GPT-4-turbo to parse and label the free-text data, identifying explicit dates (MM/DD/YYYY or YYYY/MM/DD) and inferring incident dates from relative time references (eg, “one week ago”). GPT-4-turbo was prompted to generate concise labels for AEs by recognizing key occurrences, such as medical procedures or symptoms, and condensing them into meaningful terms. For example, “She started to get a cold 2 days ago, sinus and ear pressure, nasal congestion, and cough” was labeled as “Infection,” while “Participant went to the emergency room with body aches and a fever” was labeled “Fever and body aches.” This approach enabled efficient and consistent categorization of AEs, ensuring high-quality labeled data for analysis. Further details, including the complete list of event labels and their corresponding report counts, are provided in Table S5 in Multimedia Appendix 1. Additional information on the GPT model and its use is described in Multimedia Appendix 1.

### Statistical Analysis

To assess the model’s performance, we calculated the coefficient of determination (*R*²), which quantifies how well the fitted quadratic model explains the variance in the observed data. An *R*² value closer to 1 indicates a better fit and was estimated as follows:


$$R^{2}\;=\;1\;-\;\frac{\Sigma {\left(y_{observed}\;-\;y_{fitted}\right)}^{2}}{\Sigma {\left(y_{observed}\;-\;\mu_{observed}\right)}^{2}}$$

where *y*_observed_, μ_observed_, and *y*_fitted_ represent the initial feature pattern, mean of observed data, and quadratic model output, respectively.

Kernel density estimation is a nonparametric method for estimating the probability density function of a continuous variable, providing a smooth data distribution representation. Kernel density estimation places a Gaussian kernel on each data point and sums them up to generate a continuous density estimate, avoiding parametric assumptions. To compare probability density function distributions between subgroups, we applied the Kolmogorov-Smirnov test [24]. A *P* value <.05 indicates a statistically significant difference, rejecting the null hypothesis.

To evaluate the impact of including age as a predictor, we performed a likelihood ratio (LR) test comparing the full model with age to a reduced model without it. The log-likelihoods of both models were computed, denoted as ll_full_ for the full model and ll_reduced_ for the reduced model. The LR statistic was calculated as follows:


$$LR=-2\times (ll_{reduced}-ll_{full})$$

The degrees of freedom (*df*) for the *χ*^2^ distribution were determined by the difference in the number of parameters between the full and reduced models. Using the *χ*^2^ distribution, we computed the *P* value [25] based on the LR statistic and *df*, providing statistical evidence for comparing the 2 models.

For the case study analysis, we first conducted an intraindividual assessment. For each event, we compared signal behavior during a peri-event window (7 d before and after the event, defined relative to gestational age) to a baseline period (1 mo-7 d prior). Comparisons were included only if both the baseline and peri-event periods contained at least 7 data points, ensuring sufficient data for statistical analysis. We applied an unpaired Welch *t* test [26], which does not assume equal variance, to evaluate the null hypothesis of equal means between the 2 periods. The resulting *t* statistic and *P* value quantified the magnitude and statistical significance of differences in signal behavior associated with the event window relative to baseline.

**Figure 1.**
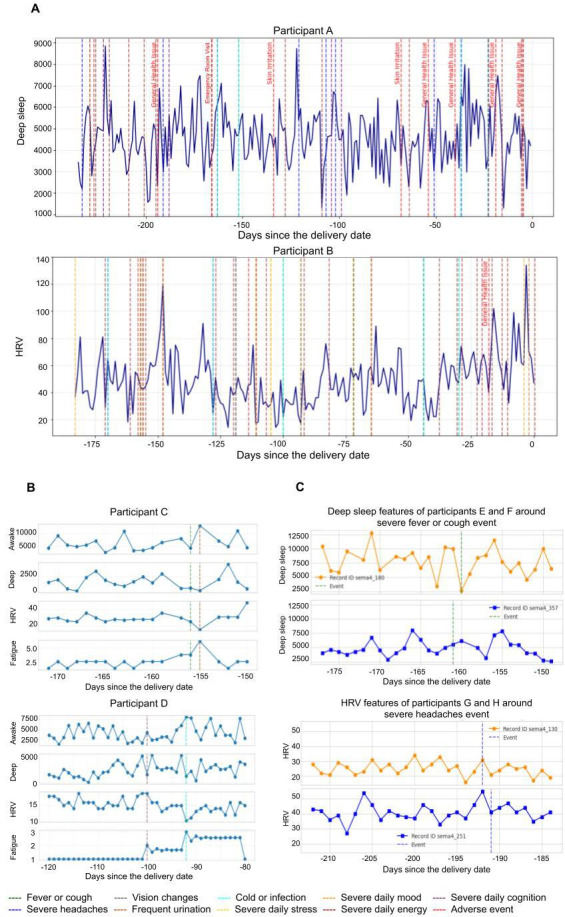
Impact of adverse events and severe symptoms on fatigue, HRV, and sleep features over pregnancy. (A) Effects of adverse or severe events on deep sleep and HRV in example BUMP participants, with adverse events shown in red next to dashed lines. (B) Examples of adverse or severe events associated with significant shifts in the distribution of features throughout pregnancy. (C) Two women experiencing the same severe symptom at the same gestational week exhibiting different sleep and HRV patterns. BUMP: Better Understanding the Metamorphosis of Pregnancy; HRV: heart rate variability.

We then performed an interindividual analysis to assess whether similar patterns were observed across individuals. Using consecutive 14-day windows spanning gestational ages from −270 days to delivery (0), we identified the occurrence of each event within each window and calculated the average *t* statistic for each individual. When multiple events occurred in close temporal proximity, events were analyzed only if their peri-event windows did not overlap; otherwise, they were treated as a single composite event. For Figure 1B, the sampling frame consisted of all participants with at least 1 adverse or severe event for which the intraindividual Welch *t* test indicated a statistically significant difference (*P*<.05) between baseline and peri-event windows. From this subset, 1 qualifying event per participant was randomly selected using uniform random sampling without replacement.

To quantify heterogeneity in responses, we defined a high-variability threshold as twice the SD of the average *t* statistic across individuals. If the absolute difference in *t* statistics between any two individuals exceeded this threshold, the event was classified as demonstrating substantial interindividual variability in associated feature changes. For Figure 1C, the sampling frame included participants who experienced the same severe symptom within the same 14-day gestational window and demonstrated statistically significant physiological changes associated with that symptom. From this eligible pool, 2 participants were randomly selected using uniform random sampling to illustrate examples of interindividual variability in physiological response patterns.

## Results

### Overview of Analysis Strategy

We performed the analysis in 4 stages. First, we replicated previously reported aggregate-level maternal wearable findings in the independent BUMP cohort, focusing on HRV, fatigue, and sleep features. Second, we quantified the extent to which these aggregate patterns reflected individual-level behavior by examining participant-specific inflection points, extrema, slopes, and longitudinal trajectories. Third, we tested whether interpersonal heterogeneity could be explained by demographic or pregnancy-complication subgroups using distributions of individual model fit. Fourth, we conducted event-centered case studies to assess whether severe symptoms and AEs could account for deviations in individual trajectories.

### Aggregated Analysis

To assess the generalizability of previously reported aggregate wearable-derived patterns in maternal health [16-18], we replicated analyses of HRV, fatigue, and sleep physiology using a larger, independent wearable dataset from the BUMP study (n=256) [21]. Aggregate trajectories over gestation were compared with those reported in prior studies, including the timing of inflection points and overall directional trends. In the following, we describe the degree to which these previously observed patterns were reproduced in the BUMP cohort and highlight areas of concordance and divergence across outcomes.

**Figure 2.**
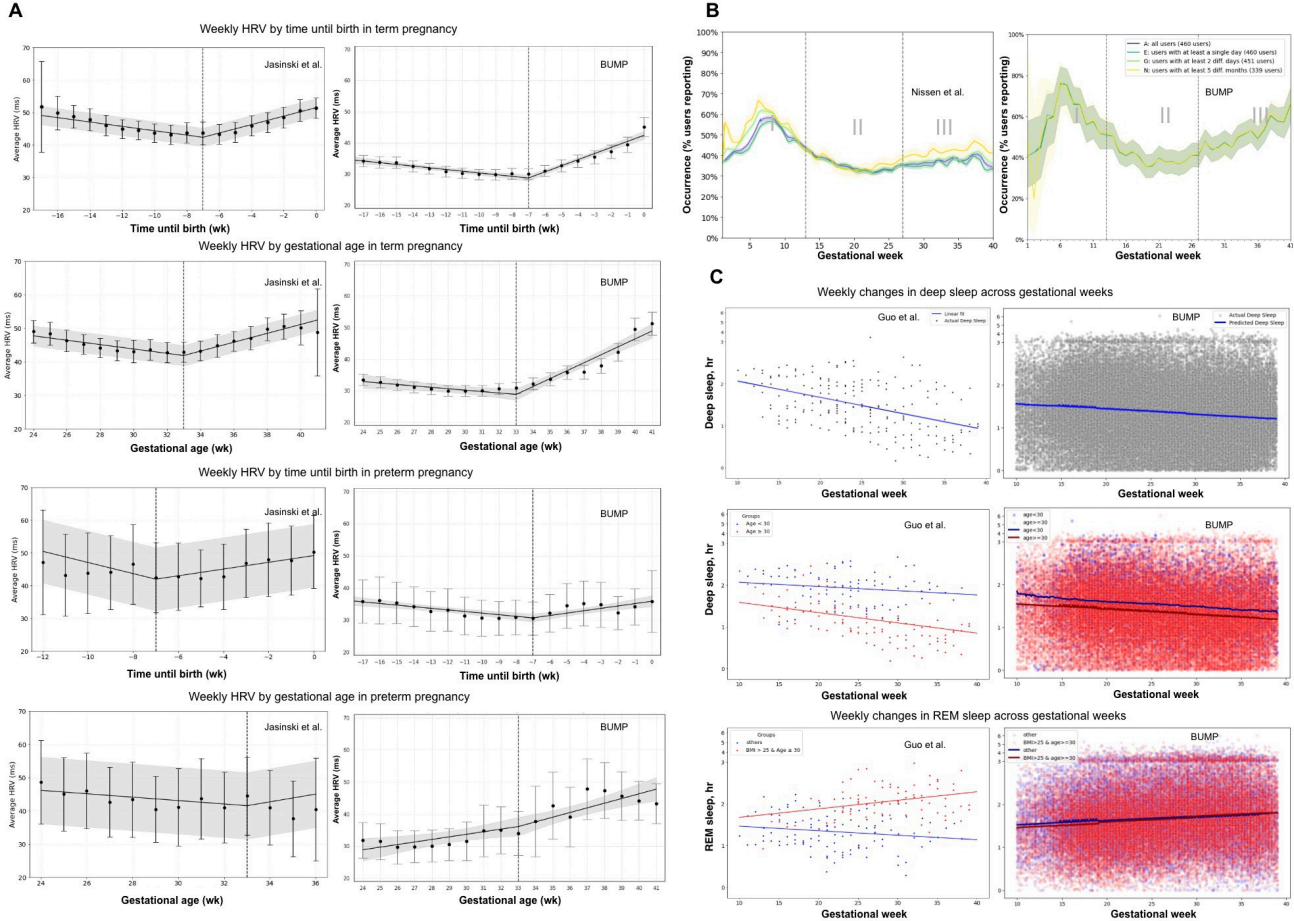
Aggregated results comparison of HRV, fatigue, and sleep patterns between BUMP [21] and prior pregnancy studies. (A) HRV trends show similar prebirth inflections, with less variance in the BUMP study. Error bars represent the 95% CI; the vertical dashed line marks the inflection point [16]. (B) Fatigue aggregate peaks and the troughs pattern in the BUMP study reproduce previously reported population-level trends, despite substantial variability in individual trajectories. Dashed vertical lines mark the pregnancy trimesters [17]. (C) Deep sleep trends differ in slope with less variation across BMI and age subgroups in the BUMP study [18]. BUMP: Better Understanding the Metamorphosis of Pregnancy; HRV: heart rate variability; REM: rapid eye movement.

In the BUMP study, the aggregated results suggest that nighttime daily HRV shows a similar inflection point around week 7 before birth in both term and preterm pregnancies and at gestational week 33 in term pregnancies, though this trend is absent in preterm pregnancies (Figure 2A). However, the weekly mean HRV values differ significantly, with the BUMP data showing a smaller weekly variance compared to Jasinski et al [16]. For fatigue, the general trends in the BUMP study reproduce those reported in Nissen et al [17], with both showing an early peak around gestational weeks 7 to 8 and a trough around week 21, although substantial deviations from this pattern are observed at the individual level, particularly in the third trimester (Figure 2B).

Regarding deep sleep (Figure 2C), both the BUMP data and Guo et al.’s findings show a negative slope for aggregate deep sleep, with Guo et al [18] reporting a slightly steeper decline across 10 to 40 gestational weeks. Our analysis further reveals substantial variability and heterogeneity in sleep data, reflected in intraclass correlation coefficients of 0.618 for deep sleep and 0.502 for rapid eye movement sleep, both exceeding the 0.5 threshold for significance. In the BUMP data, BMI and age were not clearly separable predictors, unlike Guo et al.’s findings. Segmenting age into ≥30 years versus <30 years did not significantly improve model fit (*P*=.17), suggesting that Guo et al.’s proposed associations may not be generalizable to all populations. Similarly, stratifying the data by prepregnancy BMI and age (age ≥30 and BMI>25 vs others) yielded a *P* value of 1, further questioning the generalizability of these demographic factors as predictive indicators. These findings highlight that results from wearable maternal health studies are not easily generalizable across populations due to significant heterogeneity in wearable feature patterns and individual subjective symptom experiences.

**Figure 3.**
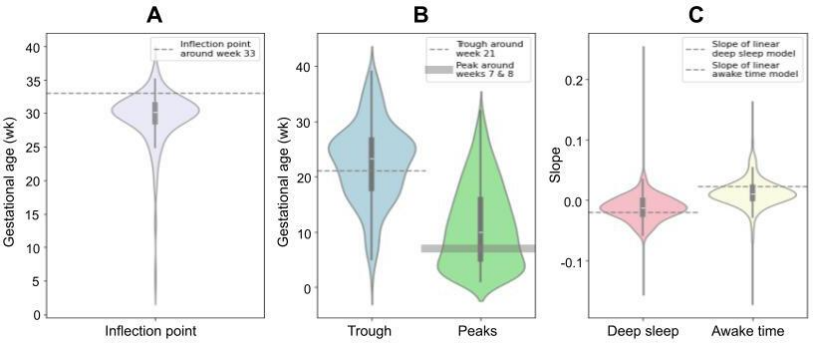
Key features extracted from wearable data during pregnancy in the BUMP study: (A) HRV, (B) fatigue, and (C) sleep. Horizontal dashed or highlighted lines represent the findings from Jasinski et al [16], Nissen et al [17], and Guo et al [18] based on aggregate results. The violin plots compare these aggregate findings with individual-level BUMP analysis, highlighting variability across participants. BUMP: Better Understanding the Metamorphosis of Pregnancy; HRV: heart rate variability.

The analysis of HRV, fatigue, and sleep metrics derived from personal DHT data during pregnancy underscores the limitations of aggregated models in capturing individual variability in maternal health. Figure 3 shows violin plots, using density curves to illustrate data distribution. Wider sections indicate higher data density, enabling intuitive comparisons of individual variability against aggregate trends. Gray lines mark key findings from each study, contrasted with BUMP dataset analyses to assess density across individual-level data.

Key comparisons include the following: For HRV, Jasinski et al [16] identified an inflection point around gestational week 33. To compare with individual-level inflection points, the BUMP data were analyzed using a quadratic model (weeks 10‐40), identifying the minimum point as the individual inflection. Similarly, aggregate fatigue trends in BUMP are consistent with Nissen et al [17], showing early peaks at weeks 7 to 8 and a trough near week 21. Individual fatigue peaks and troughs were calculated to capture variability. For sleep, Guo et al [18] used a linear mixture model to estimate slopes for aggregated deep sleep and awake time, which were compared with individual-level slopes determined by fitting linear regression models.

Figure 3 and Table S7 in Multimedia Appendix 1 show that individual-level results from BUMP, visualized through density curves, did not fully align with the published findings and revealed substantial variation. Figure 3B highlights that the median value of the gestational week in individual fatigue troughs was 23 (IQR 8) weeks, which was 2 weeks earlier than the aggregate result. Similarly, individual fatigue peaks occurred 2 weeks later than the aggregate finding, with notable variability indicated by the IQR and density spread. These discrepancies underscore the need to account for individual variability, as aggregate findings do not universally apply, even to the majority of individuals.

### N-of-1 Level Analysis

**Figure 4.**
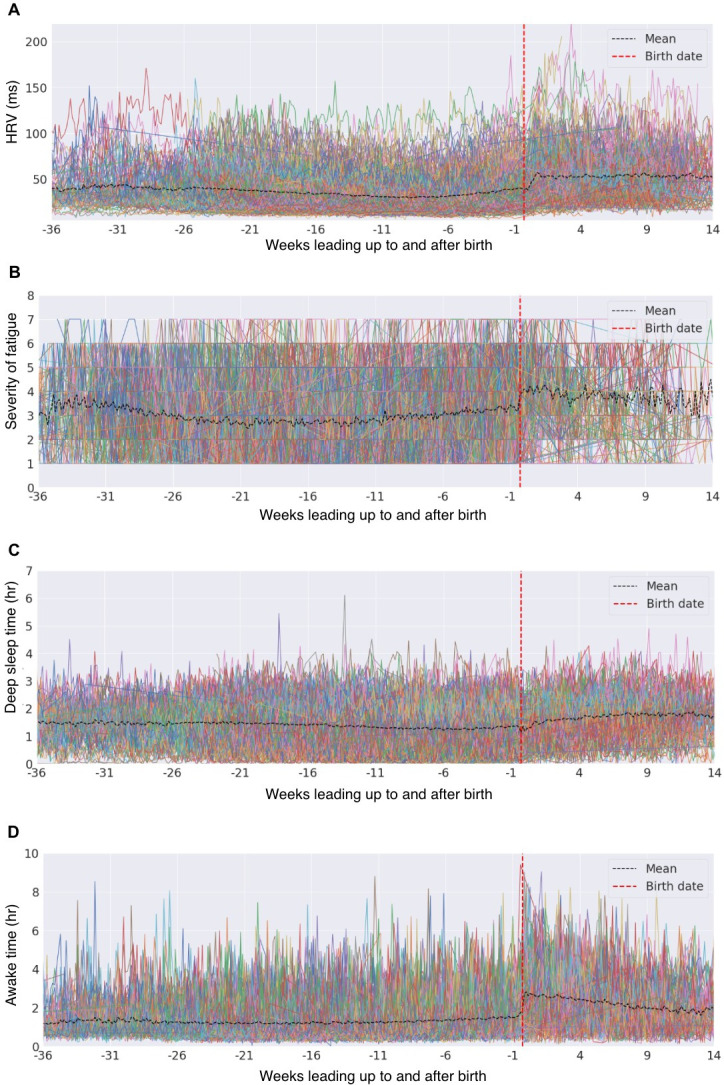
Spaghetti plots of four features from the BUMP study contrast aggregate trends with individual trajectories. Each colored line represents an individual’s (A) HRV, (B) fatigue, (C) deep sleep time, and (D) awake time, highlighting variability across gestational weeks. BUMP: Better Understanding the Metamorphosis of Pregnancy; HRV: heart rate variability.

Individual patterns in HRV, fatigue, deep sleep, and awake time were examined and compared to aggregate trends using the BUMP study data (Figure 4).

Figure 4 highlights the high variability in objective and subjective feature patterns during pregnancy, often lost in aggregate views. Individual HRV, fatigue, deep sleep, and awake time patterns vary significantly across gestational weeks. While the aggregate line (black) suggests a smooth trend, it overlooks individual complexities and key variations. Relying solely on aggregate data risks misinterpretation, especially when individual patterns deviate from the average.

**Figure 5.**
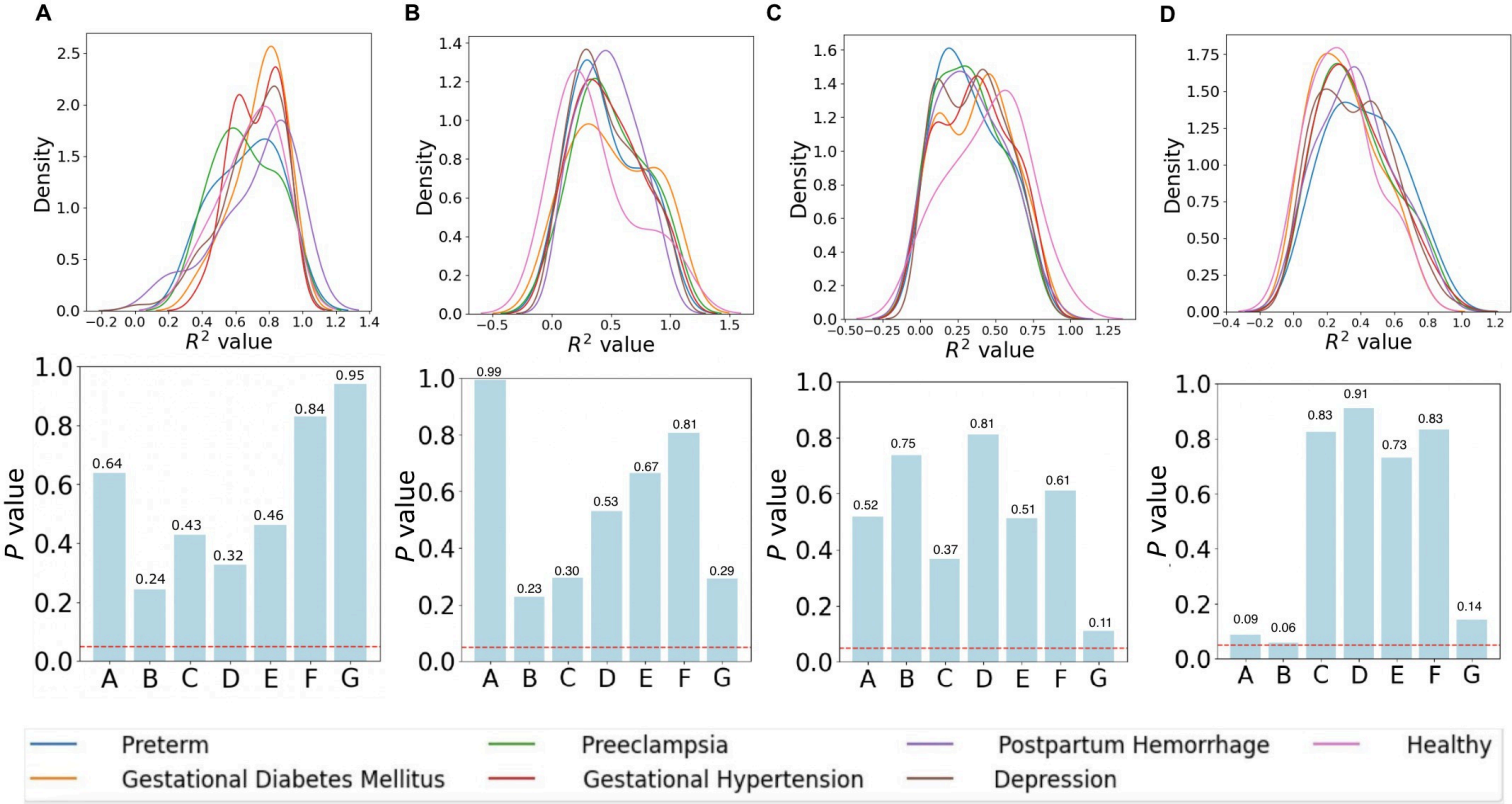
Comparison of *R*² values assessing model fit across pregnancy-related complications in the BUMP study: (A) HRV, (B) fatigue, (C) deep sleep, and (D) awake time. Top plots illustrate kernel density distributions for each group, while bottom plots display *P* values, comparing each group’s feature distribution to that of other women. The red-dashed line marks the significance threshold (*P*=.05). Kernel density plots and *P* values indicate no statistically significant differences detected in *R*² distributions across complication subgroups. BUMP: Better Understanding the Metamorphosis of Pregnancy; HRV: heart rate variability.

In Figure 5, heterogeneity is formally quantified using distributions of individual-level model fit (*R*²) and tested across subgroups using kernel density comparisons with associated *P* values. Modifying factors or confounders, such as pregnancy-related complications and demographic factors, may in part explain the observed heterogeneity in the model’s performance. However, the kernel density plots and *P* values revealed no significant differences in *R*² distributions, indicating that the model’s fit is relatively consistent across groups. Specifically, there were no significant differences in HRV, fatigue, deep sleep, or awake time trends among participants with different pregnancy-related conditions. All subgroup comparisons yielded *P* values >.05 (eg, *P*=.09, .06, .82 for awake time; *P*=.52, .75, .37 for deep sleep; *P*=.99, .23, .30 for fatigue; and *P*=.64, .24, .43 for heart rate variability) (see the lower section of Figure 5). The symmetric, overlapping kernel density plots suggest minimal variation in *R*² distributions, with no skewness or distinct clustering. These findings suggest that pregnancy-related complications and demographic factors (Figure S1 in Multimedia Appendix 1) were not associated with detectable differences in the model’s ability to fit the observed outcomes.

### Case Studies

To explore sources of heterogeneity, we analyzed survey data on severe symptoms reported by users and AEs documented in research staff notes. Key events and their precise timing were identified from unstructured AE notes using GPT-based automatic prompting, with subsequent expert review to verify the accuracy and consistency of the extracted information. We also incorporated daily severe symptoms: very low energy, impaired cognition, very low mood, and extreme stress, referred to as severe daily features. Biweekly severe symptom surveys, aligned with daily reports, covered severe fever or cough, intense headaches, severe infections, vision changes, and urinary issues. Further details are available in the *Methods* section and Multimedia Appendix 1.

Figure 1A presents examples of high-frequency AEs and severe symptoms reported during pregnancy. Among 256 individuals, 4674 events were recorded, with a median of 9 (IQR 8) and an average of 21 (SD 25.337; range 1-153) events per person. Figure 1B highlights 2 participants randomly selected from those with statistically significant shifts in feature distributions before and after severe events, as determined by an intraindividual Welch *t* test comparing baseline and peri-event periods (*P*<.05). In participant C, a low-energy event 155 days before delivery was followed by increased fatigue and awake time, along with decreased HRV and deep sleep. In participant D, a severe infection 92 days before delivery was followed by similar effects.

Figure 1C illustrates variability in physiological patterns among pregnant women experiencing the same severe symptom at the same gestational week. Participants were randomly selected from those with significant physiological responses to the same symptoms, highlighting individual variability in adaptation. For example, both participants E and F experienced severe fever, sore throat, and/or cough in the same week; yet one had a sharp decline in deep sleep, whereas the other showed a gradual increase. A linear mixed model analysis indicated substantial between-individual variability in responses across all event types. These findings underscore the need to consider both inter- and intraindividual differences when evaluating responses to similar events. Additional examples are shown in Figure S4 in Multimedia Appendix 1.

## Discussion

### Principal Findings

This study aimed to evaluate whether aggregate trends in DHT measures of HRV, sleep, and self-reported fatigue during pregnancy accurately represent individual experiences and to explore whether demographic or clinical factors explain variability among individuals. Using the BUMP dataset and N-of-1 spline modeling, we report 3 principal findings. First, aggregate trends in HRV, sleep, and fatigue were broadly consistent with those reported in prior studies but masked substantial interindividual variability. Second, N-of-1 analyses revealed that many participants deviated markedly from population averages in the timing, direction, and magnitude of changes across pregnancy. Third, neither demographic factors, pregnancy complications, nor acute health events adequately explained the observed heterogeneity, suggesting that personalized analytical approaches are necessary to accurately interpret maternal health data.

### Comparison to Prior Work

The aggregate results from these studies and the BUMP dataset demonstrate unique contours in these 3 measures of health. Notably, HRV appears to steadily decrease with gestational age, but then shows an inflection at approximately 33 weeks leading up to delivery. This inflection has been highlighted in the literature by others, suggesting a phenomenon that could reflect an individual’s physiological readiness for delivery [16,27,28]. While this could be true for some, our N-of-1 analyses demonstrate that many individuals do not experience this HRV inflection, or an inflection is experienced, but in a significantly different pattern from the average. Fatigue aggregate trends show an early peak around gestational weeks 7 to 8, followed by a gradual decline to a trough near week 21 [17]. However, individual analyses reveal considerable variations in these patterns, as shown in Figure 3. Similarly, while average sleep trends show a steady decline in deep sleep and increased awake time, especially in the third trimester [18,29], N-of-1 analyses highlight substantial interindividual differences. Some individuals experience a progressive decline in deep sleep, whereas others show minimal or even opposite changes, underscoring the complexity of sleep adaptations during pregnancy.

Physiological and subjective changes during pregnancy often differ from group averages due to external factors. While previous studies [5,18] have often relied on standard demographic exclusions or subgrouping and stratified analyses by age or pregnancy complications, these approaches miss the substantial heterogeneity in individual patterns. Using N-of-1 analyses with spline models, we captured each participant’s unique response to pregnancy-related changes, offering detailed insights into individual trajectories. Our N-of-1 analyses found no significant differences in HRV, sleep, and fatigue levels between participants with pregnancy complications and those without, suggesting that these conditions do not fully explain the observed heterogeneity. Some differences emerged across demographic factors, such as age and BMI; for instance, individuals with a BMI of 25 to 30 showed significant differences in HRV and deep sleep goodness-of-fit compared to other subgroups. However, this pattern was not observed for fatigue or awake time, indicating BMI alone is not a reliable indicator for subgrouping but warrants further exploration alongside other factors.

We further explored case studies in an attempt to isolate potential modifying factors of the examined maternal health metrics. The case studies further compounded the finding suggesting high heterogeneity of maternal health experiences. For example, in some participants, while there were associations between events, such as fever, cough, and very low energy levels, and significant increases in fatigue and awake time, there was high intraindividual and interindividual variability in responses to these events, emphasizing that pregnant individuals can exhibit diverse physiological responses to the same event at the same gestational week.

After extensive exploration into potential modifiers of HRV, sleep, and fatigue over pregnancy, we identify no clear factors explaining the observed high heterogeneity of these maternal health metrics, suggesting the necessity of personalized analytical approaches to accurately interpret maternal health data at the N-of-1 level. The significant variability among individuals highlights the complexity of deriving broadly applicable clinical insights. The absence of significant associations with the examined demographic or clinical factors should be interpreted cautiously, as limited power in smaller subgroups and unmeasured factors may contribute to the observed variability.

### Limitations

Several limitations should be considered when interpreting these findings. First, the sample size limited statistical power for subgroup analyses; small subgroup sizes may have prevented detection of meaningful associations between demographic or clinical factors and the observed variability. Second, multiple unmeasured confounders may contribute to the heterogeneity observed and were not captured in this study. Third, we note that those prior studies and BUMP differed slightly in cohort selection criteria and protocol, but such differences are unlikely to affect our conclusion, as the studies used the same device types, measurement protocols, and aggregate feature definitions for comparison.

### Conclusions

This study underscores the need for personalized and diverse assessments to capture the individuality of maternal health metrics. Moving beyond one-size-fits-all models is essential for embracing heterogeneity and tailoring insights to pregnant individuals’ unique needs. By explicitly examining N-of-1 longitudinal patterns, this study contributes to early evidence highlighting the methodological considerations necessary for safe, interpretable, and personalized maternal health monitoring. Future work should prioritize developing personalized methods that account for variability and assess the causal effects of modifying factors. Effectively interpreting personal DHT maternal health data may require human-in-the-loop approaches, where users are prompted for additional context when deviations in feature patterns are detected.

Looking forward, the findings of this study motivate the development of analytical frameworks that explicitly accommodate individual variability while leveraging population-level information. A clinically actionable N-of-1 framework would require models that explicitly borrow statistical strength from population-level data while producing calibrated, individualized inferences. One promising direction is hierarchical Bayesian time-series modeling, in which population-level dynamics inform individual trajectories without enforcing a shared average response. For example, hierarchical Dirichlet process flow-style approaches [30,31] allow each person to follow a personalized latent dynamic while sharing structure across the cohort, improving individual-level prediction under sparse or noisy observations. In pregnancy monitoring, such models could learn typical gestational regimes while estimating patient-specific deviations, enabling individualized forecasting (eg, expected HRV trajectory) and anomaly detection relative to one’s own baseline rather than a cohort mean.

## Supplementary material

10.2196/86203 Multimedia Appendix 1 Detailed methodology, data collection procedures, and extended analyses.

10.2196/86203 Checklist 1 STROBE checklist.

## Abbreviations

AEs: adverse events
BUMP: Better Understanding the Metamorphosis of Pregnancy
DHT: digital health technology
HRV: heart rate variability
LR: likelihood ratio
STROBE: Strengthening the Reporting of Observational Studies in Epidemiology
